# Apoptotic Tumor Cell-Derived Extracellular Vesicles as Important Regulators of the Onco-Regenerative Niche

**DOI:** 10.3389/fimmu.2018.01111

**Published:** 2018-05-23

**Authors:** Christopher D. Gregory, Ian Dransfield

**Affiliations:** Medical Research Council Centre for Inflammation Research at the University of Edinburgh, The Queen’s Medical Research Institute, Edinburgh, United Kingdom

**Keywords:** extracellular vesicles, apoptosis, inflammation and cancer, tumor microenvironment, angiogenesis, tissue repair and regeneration, macrophage activation, tumor biology

## Abstract

Cells undergoing apoptosis produce heterogeneous populations of membrane delimited extracellular vesicles (Apo-EVs) which vary not only in size—from tens of nanometers to several microns—but also in molecular composition and cargo. Apo-EVs carry a variety of potentially biologically active components, including small molecules, proteins, and nucleic acids. Larger forms of Apo-EVs, commonly termed “apoptotic bodies,” can carry organelles, such as mitochondria and nuclear fragments. Molecules displayed on the surface of extracellular vesicles (EVs) can contribute substantially to their size, as well as their functions. Thus far, relatively little is known of the functional significance of Apo-EVs apart from their roles in fragmentation of dying cells and indicated immunomodulatory activities. Here, we discuss EV production by dying tumor cells and consider the possible roles of Apo-EVs in a cell death-driven sector of the tumor microenvironment known as the onco-regenerative niche (ORN). We propose that tumor-derived Apo-EVs are significant vehicles of the ORN, functioning as critical intercellular communicators that activate oncogenic tissue repair and regeneration pathways. We highlight important outstanding questions and suggest that Apo-EVs may harbor novel therapeutic targets.

## Introduction: Apoptosis, and the Onco-Regenerative Niche (ORN)

**Figure 1:**
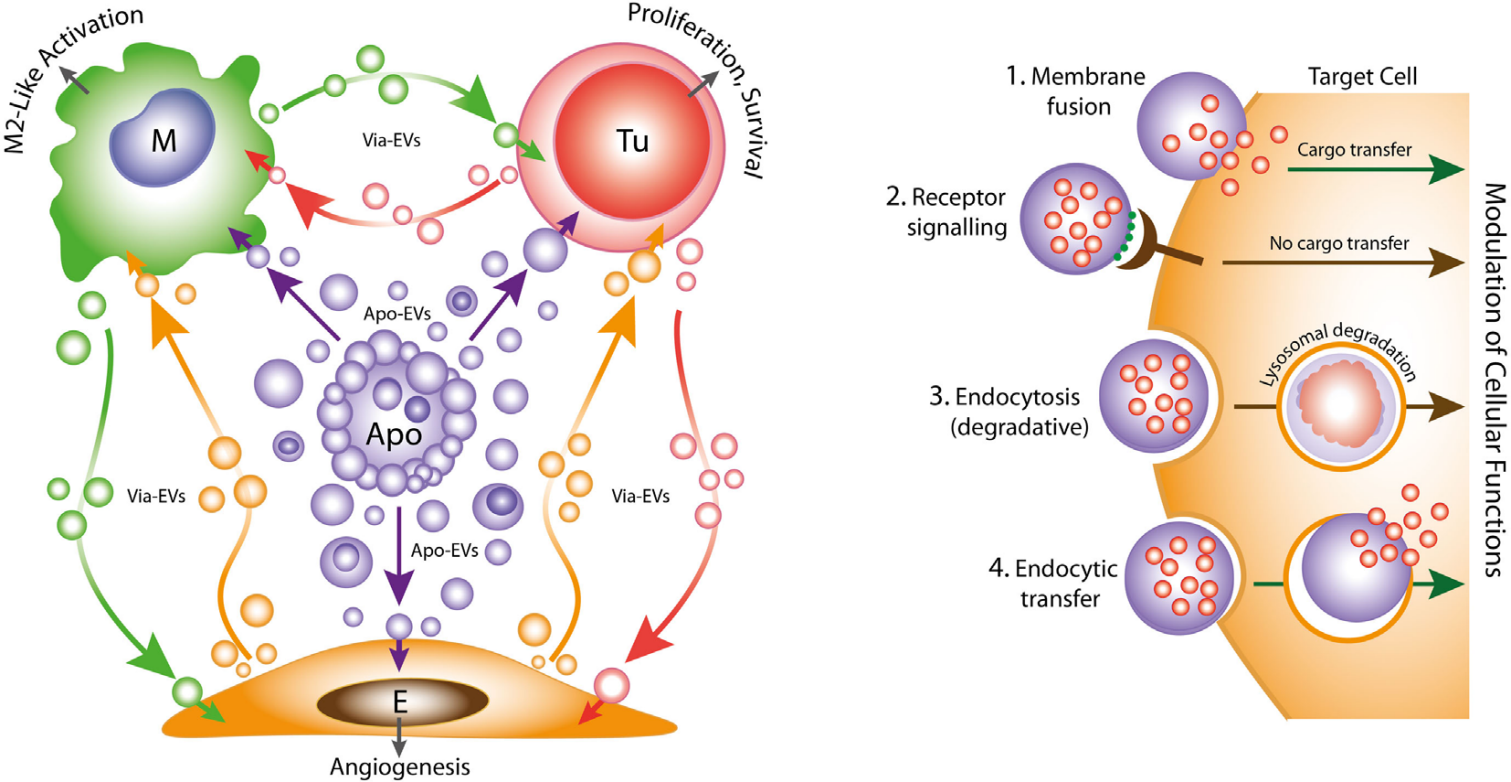
Left: oncogenic extracellular vesicle (EV) networks in the onco-regenerative niche (ORN). Schematic representation of the ORN illustrating the potential roles of Apo-EVs from dying tumor cells (Apo) in providing oncogenic signals to neighboring cells in the niche, exemplified by macrophages (M), viable tumor cells (Tu), and endothelial cells (E). We propose that Apo-EVs target such cells and modulate cellular functions, including macrophage polarization toward a reparative phenotype (M2-like activation state), promotion of tumor cell survival and proliferation, and angiogenesis. EVs from viable cells (Via-EVs) of tumor and stromal cell origin also seem likely to participate in these processes. Right: mechanisms of cell targeting and modulation by (Apo)-EVs. Examples of possible modes of interaction of Apo-EVs with target cells that may lead to modulation of cellular functions with or without transfer of intact EV cargoes (biologically active cargoes represented by small red circles). 1. Membrane fusion (receptor-dependent or -independent) permits transfer of EV cargoes to cytosolic locations. 2. Docking of EVs through receptor–ligand interaction may activate intracellular signaling pathways in the absence of cargo transfer. Ligands such as phosphatidylserine (PtdSer) (green) exposed at EV surfaces may interact directly or indirectly with target cell receptors (examples in the case of PtdSer including BAI1, TIM-4, Stabilin 2, Axl, Mer, as well as integrins αvβ3 and αvβ5). 3. Endocytic pathways (including phagocytosis) resulting in lysosomal degradation of cargoes are also likely to modulate cellular functions such as through metabolite supply and *via* receptor ligation. 4. Putative endocytic uptake of EVs without lysosomal degradation. We propose that Apo-EV cargoes are transferred intact to multiple intracellular compartments *via* this type of pathway.

In addition to its activities in developmental sculpting and adult tissue involution, apoptosis is renowned for its capacity to regulate tissue turnover and homeostasis in which, simplistically, the expansion of cell populations is balanced by regulated cell death (and *vice versa*). In cancer, this balance between cell gain and cell loss becomes dysregulated, resulting in accumulation of tumor cells and net growth of neoplastic tissues (Figure 1). By effecting controlled cell deletion, apoptosis imposes a brake on oncogenesis, a logical concept that has long been proven and is widely accepted. Indeed, inhibition of the tumor suppressor function of apoptosis led to the categorization of a new class of oncogenes—*BCL2* being the prototypic member—that could promote cell survival through suppression of apoptosis and thereby impose an oncogenic imbalance on the cell birth/cell death equation (1). Furthermore, the apoptosis effector protease, caspase-8, is mutated in multiple cancer types and the survival pathway PI3K/Akt/mTOR is dysregulated frequently in tumors (2). By contrast, pro-apoptotic regulators such as p53 and BIM (3) among others have firmly established tumor-suppressive roles for apoptosis. For these and other reasons, the capacity to evade apoptosis has become a well-accepted hallmark of cancer (4).

However, set opposite its tumor suppressor functions, the apoptosis machinery can endow dying cells with the ability to stimulate proliferation of neighboring cells, either as part of developmental programmes or in tissue repair and regeneration in adult tissues (5–9). High levels of apoptosis are commonly associated with poor prognosis in multiple cancer types (10–17) and expression of pro-apoptotic effector molecules such as active Caspase-3 and Bax can correlate with aggressive disease (18, 19). Furthermore, low-level activation of the apoptosis programme can promote genomic instability and oncogenic transformation (20).

Emerging evidence suggests strongly that both constitutive and therapy-induced apoptosis can engender pro-oncogenic responses that enhance tumor growth and cause post-therapeutic relapse (21–24). In this scenario, tumor-cell apoptosis itself promotes imbalance in the cell birth/cell death equation that ultimately favors net tumor growth. Such regenerative effects of apoptosis in the context of the tumor microenvironment led one of us to propose recently the concept of the ORN: a tumor-promoting network of tumor cells, stromal cells, and immune cells which, together with associated extracellular components, including EVs, soluble factors and matrix molecules, is orchestrated by tumor-cell apoptosis (Figure 1) (25, 26). We speculate that pervasive apoptotic tumor cell-derived signals in the ORN provide important pathways for tumor growth, metastasis and to post-therapeutic relapse. Here, we consider the potential roles of apoptotic tumor cell-derived EVs in providing such signals.

## Apo-EVs and Apoptotic Bodies

**Figure 2:**
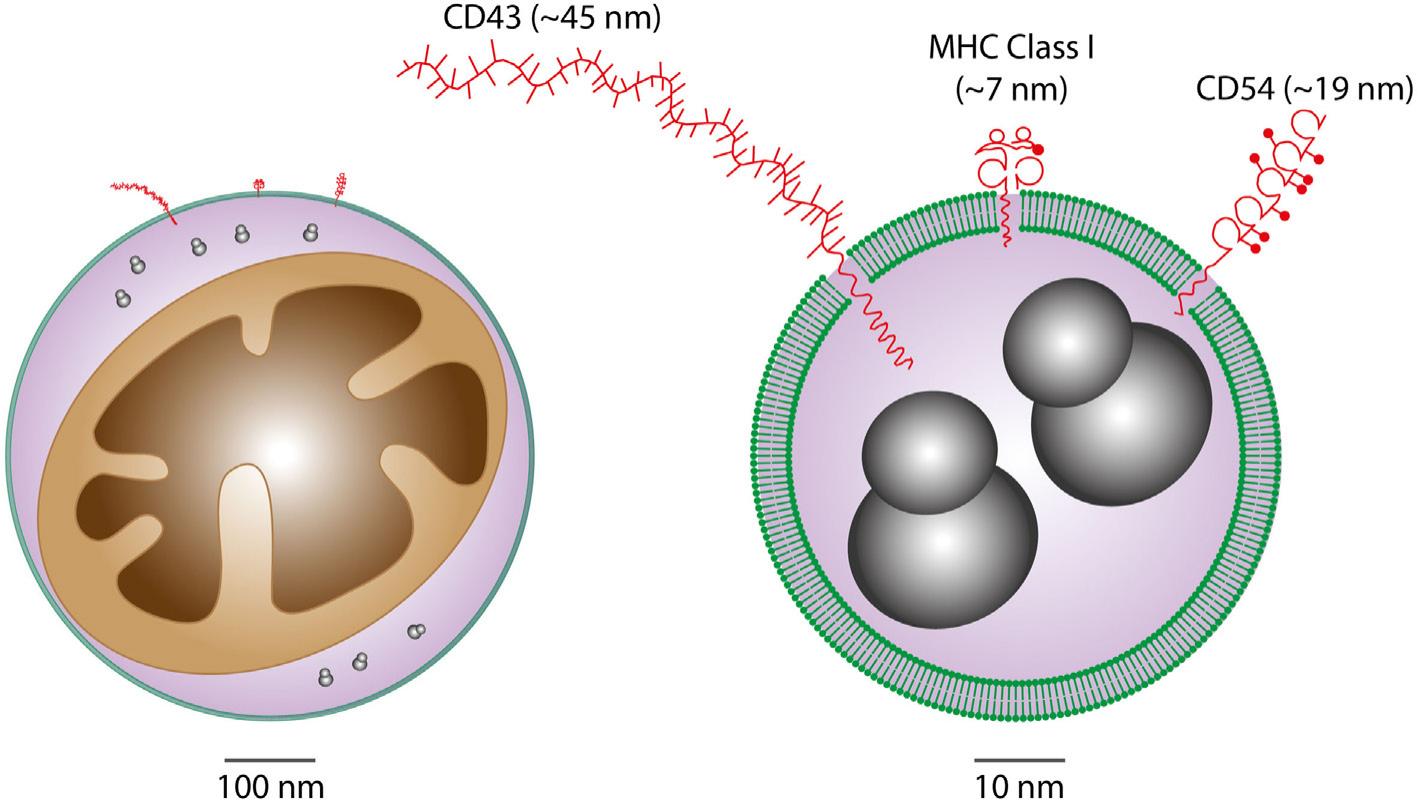
Considerations of vesicular cargoes in relation to extracellular vesicle (EV) sizes. Schematic representations of Apo-EVs of ~500 nm (left) and 50 nm (right) are shown with delimiting lipid bilayer (green), a small mitochondrion (brown), ribosomes (gray), and examples of surface proteins all drawn approximately to scale. Note the significant potential impact of surface molecule size on total vesicle size in the case of small vesicles displaying relatively large surface molecules [measurement and representation of cell surface proteins adapted from Ref. (29)].

It is becoming increasingly clear that EVs are important intercellular communication vehicles in the tumor microenvironment, shuttling an array of biologically active molecules reciprocally between tumor and non-tumor cells, modulating the development of primary tumors and metastases. We propose that Apo-EVs—as well as EVs generated in viable cells responding to their apoptotic neighbors—are important elements of the ORN (Figure 1). EV production is a well-established hallmark of apoptosis, as is surface blebbing (zeiosis) of the plasma membrane in cells responding to apoptosis stimuli. Blebs may be important precursors to Apo-EVs, but it remains unclear to what extent the process of surface blebbing is related mechanistically to the production of Apo-EVs. Here, we use the term “Apo-EV” to encompass all classes of subcellular vesicles produced as a consequence of apoptosis. These include small (~50–1,000 nm) as well as larger vesicles (1 to several microns in diameter), often referred to as “apoptotic bodies,” which harbor caspase-modified autoantigens, nuclear remnants containing condensed chromatin, and well-defined organelles, such as mitochondria and endoplasmic reticulum. Among the smaller vesicles are likely to be exosomes of endosomal pathway origin and budding plasma membrane EVs also known as microvesicles or ectosomes (27). As with all EVs, the size of Apo-EVs matters—not only in relation to what molecular and organelle cargoes can be carried but also with respect to the specific contribution of molecular cargoes, such as cell surface proteins, to overall EV size (Figure 2). While Apo-EVs are undoubtedly heterogeneous both in size and content (28), the underlying causes of this heterogeneity remain obscure.

## Apo-EV Production Mechanisms

During apoptosis, caspase-dependent cleavage, and activation of Rho-activated kinase, ROCK I alters actomyosin contractility, resulting in membrane blebbing (30). Blebbing occurs independently of altered K^+^ and Cl^−^ channel activity that results in increased K^+^ permeability and the reduction of cell volume that accompanies apoptosis. Instead, bleb formation occurs as a consequence of detachment of the plasma membrane from the actin cortex and increased hydrostatic pressure generated by the actomyosin system (31). As a consequence, there is rapid local influx of cytosolic material and “ballooning” of the membrane, resulting in bleb enlargement. Although cytoskeletal proteins are lacking in newly formed blebs, as blebbing progresses, there is reassembly of the cortical cytoskeleton underneath the membrane. Treatment of cells with the ROCK inhibitor Y-27632 reduces both the formation of apoptotic bodies and the capacity for apoptotic-cell clearance (32). Formation of small Apo-EVs may also be regulated through similar ROCK-dependent mechanisms (33), as are EVs generated by a variety of tumor cell types (34).

It has been widely assumed that the plasma membrane of apoptotic cells, apoptotic blebs, and Apo-EVs are molecularly homogeneous, displaying broadly similar changes, such as phosphatidylserine (PtdSer) exposure. However, there is evidence that apoptotic bodies exhibit loss of membrane integrity that allows limited molecular exchange (35), which may allow selective release of molecules that are able to modulate innate inflammatory mechanisms. Protein release from apoptotic bodies, particularly of nucleosomal histones, was reduced following inhibition of activity of either ROCK or myosin ATPase (35). Loss of membrane permeability may be phased as apoptosis progresses, prior to the catastrophic loss of membrane integrity during secondary necrosis. Formation of Apo-EVs and gradual loss of membrane permeability could represent a mechanism to provide transient protection of proteins from local proteolytic degradation and/or clearance, potentially allowing signals relating to cellular demise to be disseminated distally, for example to other parts of the tumor microenvironment and to metastatic sites.

Studies of the recognition and phagocytosis of apoptotic cells have revealed potentially contrasting roles for membrane blebbing and the formation of apoptotic bodies. Formation of apoptotic blebs may promote phagocytosis of apoptotic cells. Compromised apoptotic cell uptake following inhibition of blebbing (36) could be partially reversed by addition of the PtdSer opsonin, MFG-E8. Other phospholipid binding proteins such as C1q have been demonstrated to bind avidly to apoptotic blebs and C1q binds to neuronal blebs, acting to augment phagocytosis by microglia (37). ROCK-dependent high-density opsonization of apoptotic blebs could generate a topology that promotes phagocyte recognition, providing an explanation for why low-level PtdSer exposure is not sufficient to signal phagocytosis of viable cells. Thus, membrane blebbing likely facilitates maintenance of self-tolerance and suppression of antitumor immunity through direct effects on apoptotic cell clearance. Other phenomena related to vesiculation during apoptosis have also been noted recently. Following the description of apoptopodia—fine protuberances from apoptotic cells that appear to be involved in the release of larger varieties of Apo-EVs (>1 µm) (38)—EV production from certain apoptotic cell types has been observed to involve fragmentation of membrane protrusions resembling beads on a string (39). While the significance of these observations has not been fully elucidated, they provide clues as to the molecular events underlying the production of Apo-EVs and their cargo loading preferences. Intriguingly, Apo-EVs produced from beaded apoptopodia were found to be depleted of nuclear components including histones and nuclear DNA (39) that are well-known constituents of apoptotic bodies (40).

## Cargoes and Functional Activities

While EVs ostensibly of non-apoptotic cell origins have been the subject of intense research in cancer biology in recent years, the biology of Apo-EVs remains less clear. Following on from seminal work showing that glioblastoma EVs carry RNA and protein cargoes having tumor growth-promoting properties and utility as diagnostic biomarkers (41), a wealth of evidence now implicates EVs in regulating tumor growth and metastatic spread through control of angiogenesis, drug resistance, and antitumor immunity. Furthermore, the roles of EVs in intercellular communication in the tumor microenvironment are becoming better defined. Taking some recent examples, in murine melanoma, tumor cell-derived exosomes have been reported to promote the accumulation of pro-tumor macrophages *via* their ability to educate mesenchymal stromal cells which, like tumor-associated macrophages, are able to promote malignant disease *via* multiple modes, including growth factor production, suppression of antitumor immunity and angiogenesis (42). EVs from circulating tumor cells are also generated under conditions of shear flow. These EVs may play important roles in establishing the metastatic niche in the lung through interaction with the lung vasculature and rapidly accumulating myeloid cells (which phagocytose them) (43). It is noteworthy that EVs provide an intercellular signaling mechanism to transfer drug resistance to susceptible cells. For example, transfer of resistance to the multi-receptor tyrosine kinase inhibitor drug, Sunitinib can be achieved by a long, non-coding RNA (lncARSA) which acts by competing for binding to mir-34 and mir-449 to promote AXL and c-MET expression in renal cell carcinoma cells by carriage in exosomes and transfer to susceptible cells, thereby propagating resistance (44). Intriguingly, EVs isolated from cancer-associated fibroblasts are able to alter the metabolic profile of pancreatic tumor cells that interact with, and internalize them (45). Metabolic reprogramming by EVs involved inhibition of oxidative phosphorylation by mitochondria resulting in promotion of glycolysis and glutamine-dependent reductive carboxylation (46) in the recipient tumor cells. Furthermore, EVs were found to be capable of transferring multiple metabolic constituents including amino acids, lipids, citrate, and pyruvate among others, to tumor cells endowing them with the capacity to grow in nutrient-deficient media *in vitro* (45). These results strongly support the notion that EVs in the tumor microenvironment provide tumor cells with critical metabolic signals and constituents which permit growth of tumor clones under conditions of stress such as hypoxia and nutrient deprivation.

The extent to which Apo-EVs—including the larger variety, apoptotic bodies—can perform similarly diverse functions to their non-apoptotic counterparts awaits detailed clarification. However, several studies would tend to suggest that Apo-EVs represent far more than biological “waste disposal” units. We support the definition of Apo-EVs as those EVs, regardless of size or cargo, that are produced as a consequence of activation of the apoptosis effector machinery (such as executioner caspase activation) and that ultimately results in cell death. Thus, active Apo-EV production presages cell death and a major challenge for the allocation of functional properties to Apo-EVs specifically will be their discrimination from EVs produced by cells activated by other (for example, stress) pathways, including those en route to apoptosis. Like all EVs, Apo-EVs are overtly heterogeneous as illustrated by their size profile alone, which, ranges from around 30 nm to several microns (47, 48). To what extent size of Apo-EVs relates to functional properties is largely unknown, although small EVs (30–100 nm, which the authors termed “exosome-like”) produced by vascular endothelial cells downstream of caspase-3 activation were found to be distinct from their larger counterparts (microvesicles and apoptotic bodies) both in cargo composition and biological function (48). Vascular endothelial cell-derived apoptotic bodies carry histones and other nuclear proteins as well as abundant markers of organelles including mitochondria, endoplasmic reticulum, and ribosomes (48), confirming observations of apoptotic body cargoes in other systems. By contrast, the exosome-like EVs were found to be enriched in lysosomal, basement membrane and extracellular matrix proteins (48). Intriguingly, certain hallmark proteins of exosomes, including TSG101, CD9, and CD81, were missing from the exosome-like EVs whereas others, notably fibronectin, syntenin and translationally controlled tumor protein (TCTP) were present. Critically, exosome-like EVs were found to be immunogenic, in contrast to apoptotic bodies (48), confirming the presumption that the latter, as major remnants of apoptotic cells, are generally tolerogenic.

These recent studies extend earlier investigations demonstrating the segregation of nuclear components into granular and vesicular structures and extrusion from the cell in EV-like structures and apoptotic bodies (49–54). Strikingly, DNA and RNA from apoptotic cells have been described as segregating into non-overlapping vesicular entities, adding to the complexity of Apo-EV heterogeneity. It is well established that the blebs of apoptotic cell surfaces harbor antigens of common significance in autoimmune disease, including the ribonucleoproteins La and Ro and nucleosomal DNA (55). The immunogenicity of exosome-like EVs from apoptotic endothelial cells adds a further dimension to this phenomenon. Thus, the C-terminal fragment (LG3) of the basement membrane component Perlecan carried by the exosome-like EVs is highly immunogenic and may be responsible for the production of autoantibodies that can severely compromise successful renal transplantation (48). Substantial further investigations are warranted in order to clarify the differential capacity of apoptotic cells and their derived vesicles to modulate tolerance and immunity.

Besides immuno-regulatory properties, Apo-EVs have additional functional attributes based, like other EV classes, on their ability to transfer bioactive molecules to “target” cells. For example, apoptotic bodies (1–4 µm) derived from mature endothelial cells have been shown to stimulate the proliferation and differentiation of circulating endothelial progenitor cells (56). Indeed, Apo-EVs of endothelial cell origin carry a variety of biologically active components in addition to the aforementioned immunogenic Perlecan LG3, including TCTP, which can inhibit apoptosis in vascular smooth muscle cells (57). Apo-EVs may also allow the transfer of intact organelles between cells. In this context, it is noteworthy that mitochondrial transfer *via* EVs may represent an important response to stressful conditions as exemplified by the transfer of intact mitochondria from astrocytes to neurons in order to provide survival signals during the ischemic conditions of stroke (58). One of the most intriguing cargoes of Apo-EVs is genomic DNA since it has been shown that apoptotic bodies are able to mediate the horizontal transfer of DNA between somatic cells. While the details of the modes of transfer and fundamental roles of Apo-EVs (versus the remnants of apoptotic cells) have not been studied, DNA from apoptotic cells can undoubtedly be transferred to neighboring cells including tumor cells, endothelial cells, fibroblasts, and macrophages leading to apoptotic cell-derived gene expression in the recipient cells. In normal physiology, cells are protected by a DNA damage response requiring DNAse II, Chk2, and p53/p21, and deficiency in p53 and p21 can ultimately render murine embryonic fibroblasts oncogenic following transfer of DNA from apoptotic cells harboring c-myc or H-Ras oncogenes in combination with a drug resistance gene (59–61). These results have significant implications not only for genomic stability and heterogeneity of tumor cells but also for the acquisition of aberrant DNA by non-tumor cell components of the ORN, notably endothelial cells, macrophages and fibroblasts, all of which have known capacity to engulf apoptotic cells and bodies. Such genetic changes in the ORN could provide important pro-oncogenic signals even if the resultant “exogenous” gene expression is transient.

## Conclusion and Future Perspectives

While it is clear that the breakdown of apoptotic cells into membrane-bounded fragments of broad size ranges varies between different cell types, the full extent of the functional properties of Apo-EVs remains unknown. It has been reported that formation of “bite-sized” apoptotic bodies can aid in the phagocytic clearance of dying cells (36). This may be important for the apoptotic-cell clearance processes of so-called non-professional (i.e., non-macrophage) phagocytes. However, macrophages and other phagocytes have overt capacity to engulf whole apoptotic cells rapidly (62). We propose that the most important function of Apo-EVs in the context of cancer is the propagation of intercellular signals of fundamental importance to the ORN. Understanding their modes of interaction with recipient cells, their mechanisms of internalization and intracellular processing will be crucial to understanding fully the physiological and pathological attributes of Apo-EVs. To date, virtually nothing is known of these processes, although it may be expected that some clearance/engulfment mechanisms of apoptotic cells and Apo-EVs will prove to share molecular components (Figure 1). It is noteworthy in this context that PtdSer exposed on EVs is involved in their uptake by target cells expressing PtdSer receptors such as TIM-4, known for phagocytosis of apoptotic cells (63). A critical question is whether endocytosed or phagocytosed Apo-EV cargo is necessarily degraded by lysosomes, as is generally assumed. Thus, the targeting mechanisms of Apo-EVs along with the destinies of their cargoes require detailed clarification.

Pro-inflammatory extracellular vesicles (EVs) are produced by macrophages responding to ATP *via* P2X7 receptors. It has been reported recently that this results in NLPR3 inflammasome activation in human macrophages, which consequently undergo vesicle-mediated unconventional secretion of IL-1β (64). Conversely, alveolar macrophage-derived EVs have been shown to suppress airway inflammation (65). Thus, the vesicular intercommunication that results from tissue damage is likely to involve a varied mix of vesicle populations, including pro- and anti-inflammatory, derived not only from dying cells but also from their responsive neighbors or recruited phagocytes (Figure 1). Since the ORN represents a sector of the tumor microenvironment engaged in dysregulated, cell death-driven tissue repair and regeneration, it seems likely that the intercellular communications so achieved by EVs of the ORN will prove to overlap with those in healing or chronic wounds. Future work aimed at identifying the underlying mechanisms may yield novel molecular targets for both cancer and wound treatments.

## Author Contributions

Both authors planned and co-wrote the manuscript.

## Conflict of Interest Statement

The authors declare that the research was conducted in the absence of any commercial or financial relationships that could be construed as a potential conflict of interest.
